# Endozoicomonas lisbonensis sp. nov., a novel marine bacterium isolated from the soft coral Litophyton sp. at Oceanário de Lisboa in Portugal

**DOI:** 10.1099/ijsem.0.006696

**Published:** 2025-03-05

**Authors:** Daniela M. G. da Silva, Matilde Marques, Joana F. Couceiro, Elsa Santos, Núria Baylina, Rodrigo Costa, Tina Keller-Costa

**Affiliations:** 1 iBB-Institute for Bioengineering and Biosciences and i4HB-Institute for Health and Bioeconomy, Instituto Superior Técnico, University of Lisbon, Av. Rovisco Pais 1, 1049-001 Lisbon, Portugal; 2 Department of Bioengeneering, Instituto Superior Técnico, University of Lisbon, Av. Rovisco Pais 1, 1049-001 Lisbon, Portugal; 3 Oceanário de Lisboa, Esplanada Dom Carlos I s/n°, 1990-005 Lisboa, Portugal

**Keywords:** aquarium, *Endozoicomonadaceae*, host–microbe interactions, *Oceanospirillales*, octocorals, symbiosis

## Abstract

This study describes a Gram-stain-negative, rod-shaped, facultatively anaerobic bacterial species isolated from the octocoral *Litophyton* sp. inhabiting the live coral aquarium at Oceanário de Lisboa in Portugal. Four strains, NE35, NE40^T^, NE41 and NE43, were classified into the genus *Endozoicomonas* by means of 16S rRNA gene and whole-genome sequence homologies. We then performed phylogenetic, phylogenomic and biochemical analyses to examine their novel species status within the *Endozoicomonas* genus, based on comparisons with the designated novel type strain NE40^T^. The closest 16S rRNA gene relatives to strain NE40^T^ are *Endozoicomonas montiporae* CL-33^T^ (98.2%), *Endozoicomonas euniceicola* EF212^T^ (97.6%) and *Endozoicomonas gorgoniicola* PS125^T^ (97.2%). The four strains show genome-wide average nucleotide identity scores above the species level cut-off (95%) with one another and below the cut-off with all *Endozoicomonas* type strains with publicly available genomes. Digital DNA–DNA hybridization further supported the classification of the strains as a novel species, showing values below 70% when compared with other *Endozoicomonas* type strains. The DNA G+C content of NE40^T^ was 49.0 mol%, and its genome size was 5.45 Mb. Strain NE40^T^ grows from 15 to 37 °C, with 1–5% (w/v) NaCl, and between pH 6.0 and 8.0 in marine broth and shows optimal growth at 28–32 °C, 2–3% NaCl and pH 7.0–8.0. The predominant cellular fatty acids are summed feature 3 (C_16 : 1_ ω6*c* and/or C_16 : 1_ ω7*c*), summed feature 8 (C_18 : 1_ ω6*c* and/or C_18 : 1_ ω7*c*), C_16 :0_ and C_14 :0_. Strain NE40^T^ presents oxidase, catalase and *β*-galactosidase activities and can reduce nitrates to nitrites and degrade cellulose, chitin, agarose and xylan. Based on the polyphasic approach employed in this study, we propose the novel species name *Endozoicomonas lisbonensis* sp. nov. (type strain NE40^T^=DSM 118084^T^=UCCCB 212^T^).

## Data Availability

The authors confirm that all supporting data have been provided within the article. The 16S rRNA gene sequence of strain NE40^T^ is available at GenBank (National Center for Biotechnology Information) under the sequence accession number PP815993. The genome sequence is available under BioProject PRJNA1075804 and BioSample SAMN39945184. The corresponding run accession number is SRR28058719, and the assembly accession number is JBEWTB000000000.1. Table S1, on the phenotypic features of *Endozoicomonas lisbonensis* non-type strains NE35, NE41 and NE43, is available with the online version of this article.

## Introduction

The family *Endozoicomonadaceae* was officially described in 2018 by Bartz *et al*. [1] and belongs to the order *Oceanospirillales*, class *Gammaproteobacteria* and phylum *Pseudomonadota* [1,2]. *Endozoicomonadaceae* species are a widespread group of marine bacteria frequently found in symbiosis with eukaryotic organisms, including gastropods [3], sponges [4,5], corals [6,11], ascidians [12], bivalves [13] and fishes [14]. Their interactions can range from mutualistic relationships in hosts, such as corals, sponges and tunicates, to pathogenic behaviour in fishes, molluscs and echinoderms [15,16]. The genus *Endozoicomonas* is the most studied member of this family and was first described by Kurahashi and Yokota in 2007 [3]. Currently, the *Endozoicomonas* genus comprises ten validly published species: the type species *E. elysicola* [3] along with *E. montiporae* [8], *E. euniceicola* [9], *E. gorgoniicola* [9], *E. numazuensis* [4], *E. atrinae* [13], *E. arenosclerae* [5], *E. ascidiicola* [12], *E. acroporae* [6] and *E. coralli* [7]. *Endozoicomonas* bacteria are prevalent associates of marine animals, whereby their interaction with corals is particularly frequent [10]. *Endozoicomonas* spp. are found in many coral species and can represent as much as 90% of the coral bacterial community [17,18]. They organize in cluster-like structures (aggregates) inside coral tissues, which are known as cell-associated microbial aggregates [19,20]. The abundance of *Endozoicomonas* spp. and its relatives in corals is often correlated with host health status, being more abundant in healthy corals and less frequent in diseased, stressed or bleached corals [21,22]. This genus has been considered a potential ‘core symbiont’ due to its apparent intimate relationship with the coral host and may contribute to host physiology, fitness and resilience [21,23, 24]. In the present study, a polyphasic approach, combining phylogenomics, phylogenetics and phenotypic assessments, was employed to ascertain the taxonomic status of four strains (NE35, NE40^T^, NE41 and NE43) isolated from the octocoral *Litophyton* in an aquarium facility, upon which we propose the novel species termed *Endozoicomonas lisbonensis* with strain NE40^T^ as the type strain of the species.

## Isolation and ecology

The *Endozoicomonas* strains described in this study, NE40^T^, NE35, NE41 and NE43, were isolated from the tropical octocoral *Litophyton* sp. (previously *Nephthea* sp.) on 5 April 2022. Sampling took place in a 19 m^3^ tropical live coral aquarium of Oceanário de Lisboa (38° 45′ 49.3″ N 9° 05′ 37.6″ W), operational since 1998. This aquarium uses artificial seawater and operates within a closed system. It follows a 12:12 light/dark photoperiod and maintains consistent water flow through its filtration system. The life support system includes a circulation pump that enables changes in the direction of flow inside the aquarium. The water is kept at 25 °C, with a pH range of 8.15–8.24 and a salinity of 32.9–33.5 ppt. The aquarium houses a variety of marine life, including tropical octocorals, hexacorals, teleost fish, echinoderms, molluscs and crustaceans. The corals are fed every day with live *Artemia nauplii*, enriched with live *Isochrysis* microalgae, and every other day with coral V powder or *Calanus* copepods. Fragments of the octocoral were collected, placed separately into Ziploc^®^ plastic bags containing aquarium water and transported to the laboratory at Instituto Superior Técnico in Lisbon, Portugal. Sample processing took place on the same day and was performed as described in Keller-Costa *et al*. [25]. Briefly, *Litophyton* sp. fragments were rinsed with artificial seawater (ASW) (23.38 g l^−1^ NaCl, 2.41 g l^−1^ MgSO_4_.7H_2_O, 1.90 g l^−1^ MgCl_2_.6H_2_O, 1.11 g l^−1^ CaCl_2_.2H_2_O, 0.75 g l^−1^ KCl and 0.17 g l^−1^ NaHCO_3_), aseptically cut into pieces with a scalpel and homogenized in a sterile Ca^2+^- and Mg^2+^-free artificial seawater (CMFASW) (27 g l^−1^ NaCl, 1 g l^−1^ NaSO_4_, 0.8 g l^−1^ KCl and 0.18 g l^−1^ NaHCO_3_, using 1 g of coral tissue per 9 ml CMFASW w/v) with a sterile mortar and pestle. The resulting suspension was transferred into sterile 15 ml polypropylene tubes containing 2 mm sterile glass beads and further homogenized on a vortex for 1 min at maximum speed. Serial dilutions were then prepared, and 100 µl of the 10^−3^ to 10^−5^ dilutions spread plated, each in triplicates, onto 10× diluted (in ASW) Reasoner’s 2A (R2A) agar, prepared from Reasoner’s 2A broth medium (bioPlus™, bioWORLD, USA) and onto half-strength (1:2 diluted in ASW) marine agar (MA) medium. Plates were incubated at 21 °C for up to 4 weeks, and colony counts were performed weekly. Strain NE40^T^ was isolated from R2A medium and streaked to purity on the same medium, then grown in half-strength (1:2 diluted in ASW) marine broth (MB) medium (Carl Roth^®^, Karlsruhe, Germany) and preserved in 20% glycerol at −80 °C. Strains NE35, NE41 and NE43 were obtained from a second *Litophyton* sp. specimen sampled from the same aquarium on the same sampling day and isolated on half-strength MA medium, following the same procedures as for type strain NE40^T^.

## 16S rRNA gene phylogeny

Genomic DNA was extracted using the Wizard Genomic DNA Purification Kit (Promega), following the manufacturer’s instructions, and used for 16S rRNA gene and whole-genome sequencing. The quality of the genomic DNA was assessed with a NanoDrop One spectrophotometer (Thermo Fisher Scientific), and the concentration was measured with a Qubit 4.0 fluorometer (Thermo Fisher Scientific) with the dsDNA BR Assay Kit (Invitrogen). The universal primers F27 and R1492 [26] were used to amplify near-complete 16S rRNA gene fragments, which were subsequently Sanger-sequenced using both primers, as described by Esteves *et al.* in [27] and the consensus sequences generated.

**Fig. 1.**
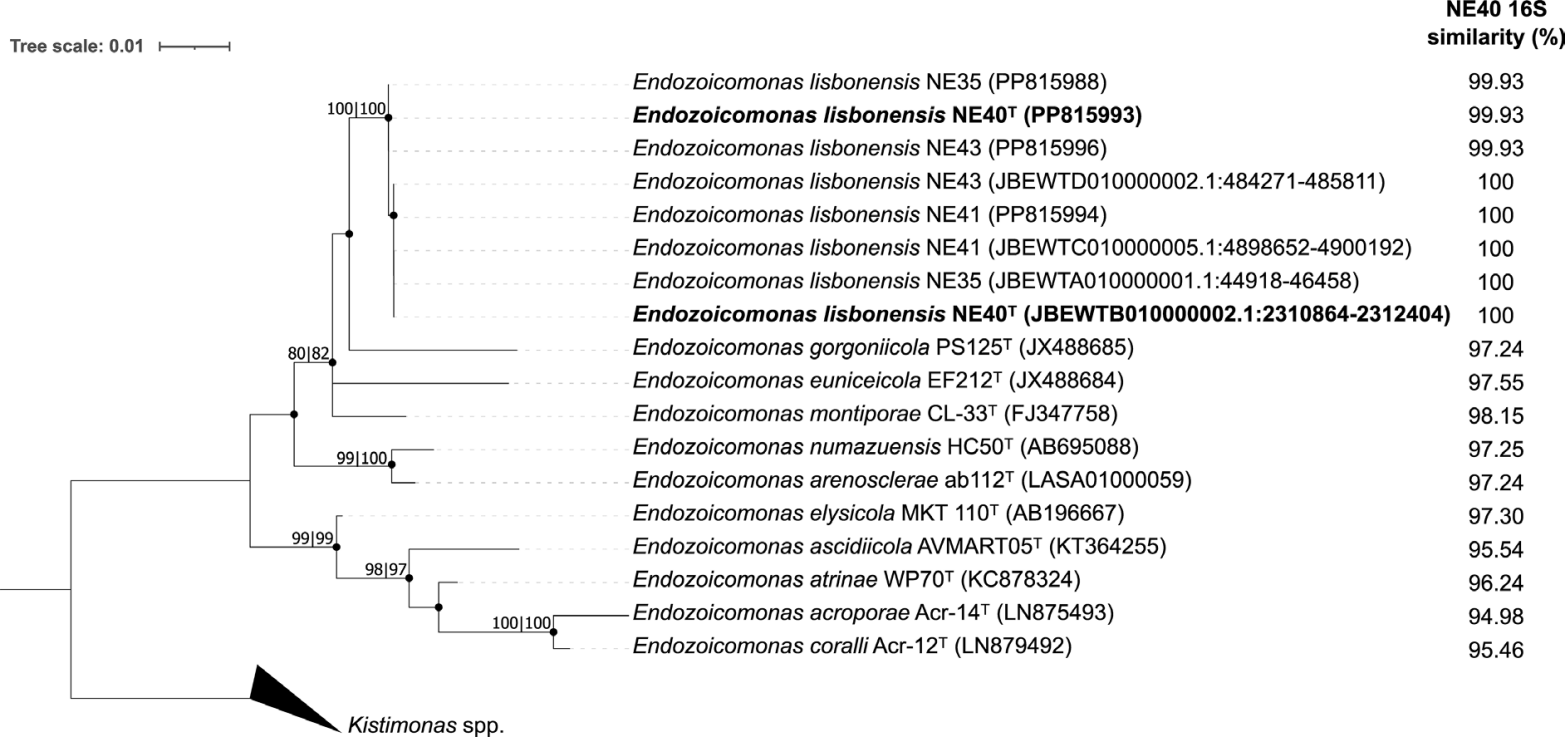
16S rRNA gene-based phylogenetic tree of the *Endozoicomonas* genus. The tree depicts ten 16S rRNA gene sequences from formally described *Endozoicomonas* type strains, plus the near-complete (≥1362 bp) Sanger sequences and the complete (1541 bp) 16S rRNA gene sequences retrieved from the genome assemblies of strains NE40^T^, NE35, NE41 and NE43. The tree was generated using the ML algorithm and the Kimura two-parameter model with a discrete gamma distribution to model evolutionary rate differences among sites [eight categories (+G, parameter=0.1577)] and invariant sites [(+I), 32.57% sites]. The tree is drawn to scale (scale bar in the figure). The tree with the highest log likelihood (3395.23) is shown. Partial deletion was used, and all positions with less than 85% site coverage were eliminated. There were a total of 1373 positions in the final dataset. Filled circles indicate that the respective nodes were also recovered from the NJ tree. Bootstrap values above 70% for each algorithm (ML/NJ) are displayed adjacent to the respective nodes. 16S rRNA gene GenBank accession numbers (or NZ genome locus tags) are given in parentheses after the strain names. 16S rRNA gene similarity values between NE40^T^ and *Endozoicomonas* type strains are shown on the right. These values were estimated by calculating pairwise distances with the Kimura two-parameter model within mega v.11, using the 16S rRNA gene sequence (locus tag: JBEWTB010000002.1:2310864-2312404) of the NE40^T^ genome as reference. Three 16S rRNA gene sequences of type strains from the closely related genus *Kistimonas* were included as an outgroup to root the tree.

The phylogenetic position of the novel species was determined by aligning the 16S rRNA gene sequences of strains NE40^T^, NE35, NE41 and NE43 (both the Sanger sequence and a representative sequence retrieved from the genomes) with 16S rRNA gene sequences of all *Endozoicomonas* type strains available on the List of Prokaryotic Names with Standing in Nomenclature. mega version 11 was used to reconstruct phylogenetic trees (Fig. 1), employing the maximum likelihood (ML) and neighbour-joining (NJ) algorithms. The clade robustness of the obtained trees was assessed by performing a bootstrap analysis using 1000 replicates. The 16S rRNA gene sequence similarity between the four novel strains and all *Endozoicomonas* type strains was estimated by calculating pairwise distances with the Kimura two-parameter model within mega v.11, using the 16S rRNA gene obtained from the NE40^T^ genome sequence as the reference sequence. The resulting phylogenetic trees confirmed the closeness of strain NE40^T^ to the type strains *E. gorgoniicola* PS125^T^ (=DSM 26534^T^; 97.2% sequence similarity), *E. euniceicola* EF212^T^ (=DSM 26535^T^; 97.6%) and *E. montiporae* CL-33^T^ (=LMG 24815^T^; 98.2%). The phylogenetic inference, together with the 16S rRNA gene similarity values between strain NE40^T^ and its closest type strains, indicates that this strain, along with strains NE35, NE41 and NE43, may represent a novel species within the genus *Endozoicomonas*. To ascertain the position of strains NE40^T^, NE35, NE41 and NE43 as novel *Endozoicomonas* species, a series of genome-wide comparative analyses were conducted.

## Genome features

The draft genomes of strains NE40^T^, NE35, NE41 and NE43 were generated at the DOE Joint Genome Institute (JGI) using the Pacific Biosciences (PacBio) sequencing technology as previously described in detail [28]. Briefly, a >10 kb PacBio SMRTbell™ library was constructed and sequenced on the PacBio Sequel platform. Reads >5 kb were assembled with Flye (2.8.3) using default settings [29]. The final draft assembly of strain NE40^T^ contained three contigs in three scaffolds (N50=5 065 071 bp), totalling 5 450 835 bp in size. The input read coverage was 201.9×.

The genome size of strain NE40^T^ is 5.45 Mb, which is similar to that of strains NE35, NE41, NE43 and *E. montiporae* CL-33^T^, but smaller than most of the other *Endozoicomonas* species (Table 1). Strain NE40^T^ and the other three novel strains displayed G+C contents of 49.0 mol%, which is within the range previously described for the genus *Endozoicomonas* (Table 1).

**Table 1.** General genome features of strains NE40^T^, NE35, NE41 and NE43 and of all (*n*=9) *Endozoicomonas* type strains with publicly available genomes Data were retrieved from JGI’s Integrated Microbial Genomes & Microbiomes platform [42,43].

Strain	Isolation source	Genome size (Mb)	Gene count	G+**C**(mol%)	Coding sequencecount assembled	RNA count assembled
NE40^T^	*Litophyton* sp. (octocoral)	5.45	4997	49.0	4840	139
NE35	*Litophyton* sp. (octocoral)	5.46	5007	49.0	4850	139
NE41	*Litophyton* sp. (octocoral)	5.47	5030	49.0	4875	137
NE43	*Litophyton* sp. (octocoral)	5.45	4991	49.0	4834	139
*E. acroporae* Acr-14^T^	*Acropora* sp. (hexacoral)	6.05	5363	49.16	5221	132
*E. arenosclerae* ab112^T^	*Arenosclera brasiliensis* (marine sponge)	6.42	5984	47.67	5821	163
*E. ascidiicola* AVMART05^T^	*Ascidiella* sp. (ascidian)	6.13	5067	46.70	4940	127
*E. atrinae* WP70^T^	*Atrina pectinate* (bivalve)	6.51	6300	47.87	6198	102
*E. elysicola* MKT 110^T^	*Elysia ornata* (gastropod)	5.61	4714	46.75	4601	113
*E. euniceicola* EF212^T^	*Eunicea fusca* (octocoral)	6.52	5567	48.0	5398	138
*E. gorgoniicola* PS125^T^	*Plexaura* sp. (octocoral)	6.34	6074	48.0	5830	135
*E. montiporae* CL-33^T^	*Montipora aequituberculata* (hexacoral)	5.43	5106	48.46	4940	166
*E. numazuensis* HC50 ^T^	*Haplosclerida* (marine sponge)	6.34	5516	47.02	5317	199

Whole-genome average nucleotide identity (ANI) values and digital DNA–DNA hybridization (dDDH) probabilities were calculated between strain NE40^T^; strains NE35, NE41 and NE43; and the already described species of the *Endozoicomonas* genus (Table 2). Strain NE40^T^ displayed ANI scores below the species level cut-off (95%) against all already described *Endozoicomonas* type strains. NE40^T^ shared 89.27, 85.79 and 82.33% ANI with *E. gorgoniicola* PS125^T^, *E. euniceicola* EF212^T^ and *E. montiporae* CL-33^T^, respectively. The dDDH between strain NE40^T^ and all described *Endozoicomonas* species with complete genomes available was lower than 70% in all cases, varying between 23.20 and 38.70% (Table 2). Among the four novel strains, NE40^T^, NE35, NE41 and NE43, the ANI values were over 99.99%, and the dDDH was 100%, confirming that these strains belong to the same species. Both ANI and dDDH values establish the status of strains NE35, NE40^T^, NE41 and NE43 as members of a novel species within the genus *Endozoicomonas*.

**Table 2.** Whole-genome similarity between strain NE40^T^; strains NE35, NE41 and NE43; and all (*n*=9) *Endozoicomonas* type strains with publicly available genomes The tool Genome-to-Genome Distance Calculator (GGDC) was used to calculate the probability of dDDH being over 70% (using the recommended formula d4: sum of all identities found in HSPs divided by overall HSP length), and the G+C difference (mol%) for all genomes presented here. Whole-genome ANI and alignment fraction (AF) values were calculated for all genomes on KBase with FastANI. The web service JSpeciesWS was used to calculate all ANI values based on blast+ (ANIb).

	GGDC			FastANI (KBase)		ANI calculation based on blast+ (JSpecies)			
**Strain**	**dDDH**	**Prob. dDDH≥70%**	**G+C difference (mol%)**	**ANI (%)**	**AF (%)**	**ANIb (%)**	**Aligned (%)**	**Aligned (bp)**	**Total (bp)**
NE35	100.00	98.29	0.01	99.9902* 99.9951†	99.28* 98.96†	99.99	99.23	5 408 948	5 450 835
NE41	100.00	98.29	0.01	99.9911* 99.9925†	99.17* 98.79†	99.99	99.39	5 417 734	5 450 835
NE43	100.00	98.29	0.0	99.9911* 99.9926†	99.83* 99.78†	99.99	99.85	5 442 508	5 450 835
*E. acroporae* Acr-14^T^	23.70	0.0	0.14	77.4732* 77.4538†	9.58* 9.75†	70.61	25.84	1 408 401	5 450 835
*E. arenosclerae* ab112^T^	23.30	0.0	1.37	78.3859* 78.3645†	14.48* 13.31†	71.58	33.62	1 832 312	5 450 835
*E. ascidiicola* AVMART05^T^	24.00	0.0	2.33	77.9278* 77.9444†	9.64* 9.83†	70.19	26.69	1 454 818	5 450 835
*E. atrinae* WP70^T^	23.20	0.0	1.09	77.3871* 77.6347†	10.30* 9.23†	70.47	28.83	1 571 692	5 450 835
*E. elysicola* MKT 110^T^	24.20	0.01	2.28	78.3841* 78.3395†	8.98* 8.46†	70.23	29.37	1 600 692	5 450 835
*E. euniceicola* EF212^T^	32.20	0.25	0.77	85.7863* 85.8403†	61.56* 52.07†	85.21	53.61	2 922 134	5 450 835
*E. gorgoniicola* PS125^T^	38.70	1.95	0.78	89.2667* 89.2077†	63.60* 55.73†	88.16	56.93	3 103 061	5 450 835
*E. montiporae* CL-33^T^	24.50	0.01	0.56	82.3314* 82.4948†	56.66* 56.41†	80.47	56.27	3 066 994	5 450 835
*E. numazuensis* HC50^T^	25.60	0.01	2.01	78.9556* 78.7173†	13.71* 12.77†	71.80	32.23	1 756 885	5 450 835

* Genome of NE40T as the query genome.

† Genome of NE40T as the reference genome.

**Fig. 2.**
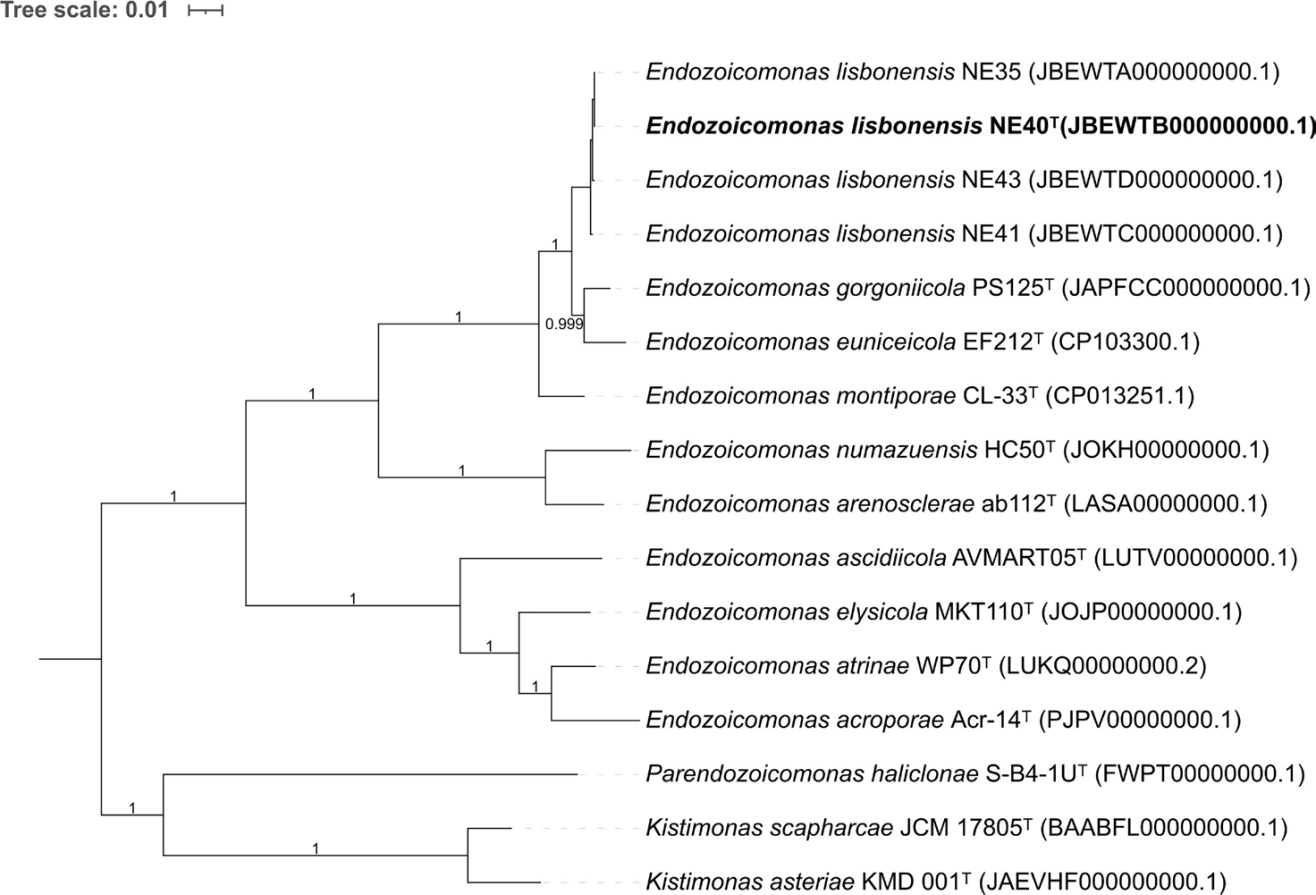
Phylogenomic tree of the *Endozoicomonas* genus, based on 49 core, universal genes defined by COGs of proteins. The genome sequences of nine *Endozoicomonas* type strains plus the genomes of strains NE40^T^, NE35, NE41 and NE43 were included in the analysis. The FastTree2 algorithm, with the ML method and Jukes–Cantor evolutionary model with a category approximation (CAT model), was used with 1000 repetitions to estimate bootstrap support values [32] within the KBase online platform (www.kbase.us) [30]. Three genome sequences of type strains from the closely related *Endozoicomonadaceae* and the genera *Kistimonas* and *Parendozoicomonas* were used as an outgroup to root the tree.

The phylogenomic analysis of the novel species and the nine other *Endozoicomonas* species (all type strains with genome sequences available) was performed with the SpeciesTreeBuilder version 0.1.4 application with the function ‘Insert Set of Genomes into Species Tree’ of the DOE Systems Biology Knowledgebase (KBase) online platform (www.kbase.us) [30]. The genomes were previously annotated with Prokka [31] and used as input within KBase. Sequences were aligned based on a multiple sequence alignment using a set of 49 core clusters of orthologous groups (COGs) of proteins [30]. The FastTree2 algorithm [32] was used to generate an ML phylogenomic tree (Fig. 2).

## Morphological characteristics

**Fig. 3.**
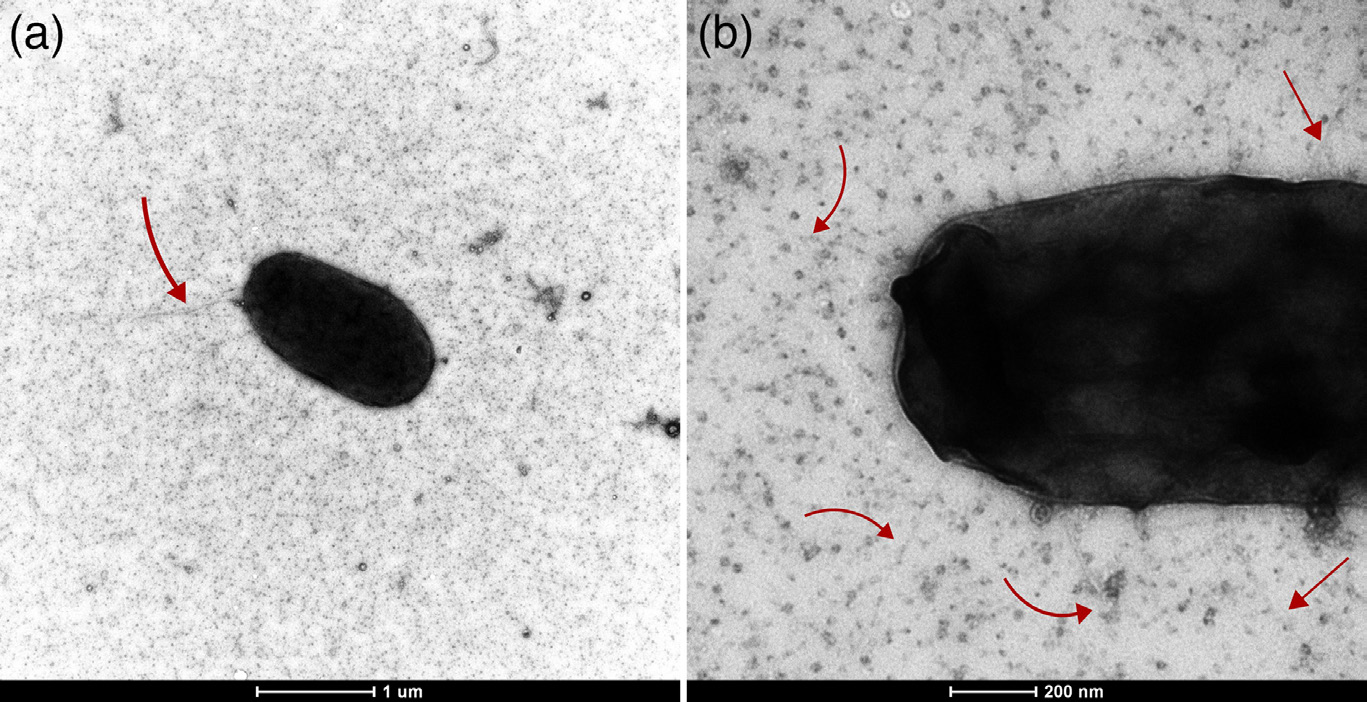
TEM showing the general morphology of negatively uranyl acetate-stained cells of strain NE40^T^. (a) General morphology and the presence of a single flagellum; magnification, 5000×. (b) The presence of pili attached to the cell surface; magnification, 60 000×.

Strains NE40^T^, NE35, NE41 and NE43 were tested for their Gram reaction following the standard methods [33]. Staining results and cell morphology were observed under an Axioplan microscope (Zeiss) at ×1000 magnification. Cell motility was tested using the hanging drop method [34]. Ultrastructure appendages (flagella and pili) were observed in strain NE40^T^ using transmission electron microscopy (TEM) after negative staining with uranyl acetate (Fig. 3). TEM visualization of the NE40^T^ cells was also used to further investigate the species’ cell morphology and size. Cells were Gram-stain-negative, rod-shaped, 0.5–1.0 µm wide and 1.5–3.0 µm long and motile. TEM analyses revealed the presence of a single flagellum as well as multiple pili and vesicles attached to the cell surface. After 3 days of incubation at 30 °C on MA, strains NE40^T^, NE35, NE41 and NE43 form translucent cream, circular, convex, smooth colonies with an entire margin and 0.5–1.5 mm in diameter (Tables 3 and S1, available in the online Supplementary Material).

**Table 3.** Phenotypic features of strain NE40^T^, its closest type strain relatives and the type species of the *Endozoicomonas* genus Strains: 1, NE40^T^; 2, *E. gorgoniicola* PS125^T^ [9]; 3, *E. euniceicola* EF212^T^ [9]; 4, *E. montiporae* CL-33^T^ [8]; 5, *E. elysicola* MKT110^T^ [3]. Strains 1–5 are motile and present catalase and oxidase activities. Strains 1–5 do not produce indole, and neither show arginine dihydrolase activity, gelatine hydrolysis (protease) or urease activity, nor do they utilize l-arabinose. +, Positive; w, weakly positive; v, variable; −, negative; nd, no data available; A, aerobe; FAN, facultative anaerobe; S, susceptible (>3 mm inhibition zone); MS, moderately susceptible (1–3 mm inhibition zone); R, resistant (<1 mm inhibition zone). Please note that results obtained for strains NE35, NE40 and NE43 are presented in Table S1.

Characteristic	1	2	3	4	5
Colony pigmentation	Translucent cream	Creamy white	White	Beige	Beige
Colony morphology	Circular, smooth and convex with entire margins	Circular, convex	Circular, convex	Circular, convex with entire edges	Circular, convex, smooth and shiny with entire margins
Colony diameter (mm)	0.5–1.5	0.5–1.0	0.2–0.5	1.0–2.0	4.0–5.0
Cell length (μm)	1.5–3.0	1.7–2.5	1.7–2.6	1.0–3.0	1.8–2.2
Cell diameter (μm)	0.5–1.0	0.4–0.9	0.6–0.9	0.5–0.7	0.4–0.6
Temperature range for growth (°C) (optimum)	15–37 (28–32)	15–30 (22–30)	15–30 (22–30)	15–35 (25)	4–37 (25–30)
pH range for growth (optimum)	6.0–8.0 (7.0–8.0)	7.0–9.0 (8.0)	7.0–8.0 (8.0)	6.0–10.0 (8.0)	nd
NaCl concentration for growth(%, w/v) (optimum)	1.0–5.0 (2.0–3.0)	1.0–4.0 (2.0–3.0)	1.0–4.0 (2.0–3.0)	1.0–3.0 (2.0–3.0)	nd
Relation to O_2_	FAN	FAN	FAN	A	A
Motility	+	+	+	+	+
Biofilm formation	w	nd	nd	nd	nd
Reduction of nitrate	+	−	−	+	+
Oxidase	+	+	+	+	+
*β*-Galactosidase	+	w	w	−	−
*β*-Glucosidase (aesculin hydrolysis)	+	w	w	+	+
Utilization of:					
Starch	−	−	−	−	nd
d-Glucose	−	+	+	+	nd
d-Mannose	−	+	−	−	−
d-Mannitol	−	+	−	−	−
* N*-Acetyl-glucosamine	v	+	+	+	+
d-Maltose	v	+	v	+	+
d-Gluconic acid	−	−	−	−	nd
Antibiotic resistance					
Ampicillin Gentamicin Chloramphenicol	SRS	SMSS	SSS	SSS	ndndnd

## Physiological and biochemical analyses

The temperature, pH and salinity growth intervals were determined following similar procedures as described for the closest *Endozoicomonas* species [8,9] and as explained earlier [35], using pre-inocula grown for 3 days at room temperature (RT) in half-strength MB. The temperature range was assessed in half-strength MB at 4, 10, 15, 18, 22, 25, 28, 30, 32, 34, 37 and 40 °C incubated at 100 r.p.m. orbital shaking for a maximum of 14 days or until detection of visible growth. The ability to grow between pH 2.0 and 10.0 in MB (with increments of 1.0 adjusted by the addition of 5 M HCl or 10 M KOH to MB) was also tested. Salinity tolerance was assessed in modified MB containing 2.41 g l^−1^ MgSO_4_, 1.90 g l^−1^ MgCl_2_, 1.11 g l^−1^ CaCl_2_, 0.75 g l^−1^ KCl, 0.203 g l^−1^ KHCO_3_, 5 g l^−1^ of peptone and 1 g l^−1^ of yeast extract and supplemented with different concentrations of NaCl from 0–10% with increments of 1%. Strains were mostly able to grow from 15 to 37 °C (optimum 28–32 °C), between pH 6.0 and 8.0 (optimum 7.0–8.0) and between 1 and 5% (w/v) NaCl (optimum 2–3%) (Tables 3 and S1). Oxygen requirements of the tested strains were assessed in static glass tubes containing thioglycolate broth with resazurin (Millipore) dissolved in CMFASW, as earlier described [35]. Moreover, the ability to grow in microaerophilic (6% O_2_, 7.1% CO_2_, 3.6% H_2_ and 83.3% N_2_) and anaerobic (10% CO_2_, 5% H_2_ and 85 % N_2_) conditions (achieved with an atmosphere generation system, Anoxomat^®^, Advanced Instruments) was also tested on half-strength MA plates incubated at 30 °C for 14 days or until visible growth was observed. All four strains, NE40^T^, NE35, NE41 and NE43, were found to be facultative anaerobes, able to grow in aerobic, microaerophilic and anaerobic conditions (Tables 3 and S1).

Strains were tested for catalase activity using the slide (drop) method by smearing a loopful of freshly grown colonies on a microscope glass slide and dripping 10 µl of 3% (v/v) hydrogen peroxide over the cells [36]. The immediate formation of effervescent bubbles indicated that all stains had catalase activity. Biofilm formation was assessed in static glass tubes [37]. Briefly, glass tubes were equipped with 5 ml of MB inoculated with 50 µl of bacterial cell culture and incubated standing without agitation for 7 days at 30 °C. A thin and fragile biofilm was observed at the air–liquid interface for NE40^T^ and the other three strains. Degradation of cellulose, starch, xylan, agarose and chitin was tested on MA 1:5 (diluted in ASW) agar plates supplemented with 0.5% (w/v) of the respective polysaccharide. In these assays, the agar plates were spot-inoculated with 10 µl of pre-inocula (grown for 3 days at RT in half-strength MB) and incubated for 21 days at RT. After 7, 14 and 21 days, the plates were observed for the presence of degradation halos. To visualize the halos, the agar plates were flooded with 2 ml of a specific stain, depending on the type of medium: Lugol’s iodine solution was used as a stain to visualize degradation halos on agarose-, starch- and chitin-based media, while a 0.1% Congo Red solution was used for cellulose- and xylan-based media. The excess stain was removed, and after 30 min of staining, the plates were checked for the presence of degradation halos. Proteolytic activity was evaluated on MA 1:5 plates supplemented with 0.5 or 1% (w/v) of skimmed milk powder solution, and this assay was performed in the same conditions as the polysaccharide degradation assays. Protease plates were observed directly (with no stain) for the presence of translucent halos around the colonies. Strains NE40^T^, NE35, NE41 and NE43 were able to degrade agarose, cellulose, chitin and xylan, but not starch, and showed no proteolytic activity. Siderophore production was tested on MA plates containing chrome azurol S (CAS) solution. The CAS solution was prepared as described by Schwyn and Neilands [38] and mixed with MA in a 1:10 ratio before autoclaving. Five microlitres of 5-day-old pre-inocula were spot-inoculated onto the agar plate, and the plates were incubated at 30 °C for 2 weeks, after which the formation of halos around the colonies, as well as a change of medium colour from blue to orange, was observed for NE40^T^ and the other three strains, indicating the production of siderophores [39].

The biochemical profile of the novel strains, NE40^T^, NE35, NE41 and NE43, was further analysed with the commercial analytical profile index (API^®^, bioMérieux). Nitrate reduction; indole production; glucose fermentation; hydrolysis of aesculin and gelatine; activities of arginine dihydrolase, *β*-galactosidase (para-nitrophenyl-*β*-d-galactopyranoside) and urease; and the utilization of glucose, arabinose, mannose, mannitol, *N*-acetylglucosamine, maltose, gluconate, caprate, adipate, malate, citrate and phenylacetate were tested by employing commercial API 20 NE galleries for Gram-stain-negative, non-enteric and non-fastidious bacteria. The preparation of the galleries was done as described by the manufacturer, with modification on the bacterial cell suspension of the *Endozoicomonas* strains, which were prepared in 2% (w/v) NaCl instead of 0.85%. Additionally, the API^®^ 20 NE AUX^®^ medium from the kit was modified by adding 800 µl of a 20% NaCl (w/v) solution to 7 ml of the commercial kit solution, achieving a final NaCl concentration of 2.1%. The API galleries were incubated at 30 °C for 72 h. Oxidase activity was determined using commercial oxidase strips (Millipore, BioChemika and Sigma-Aldrich). The results are summarized in Tables 3 and S1, and a detailed list of results is presented in the species description below.

Strains NE40^T^, NE35, NE41 and NE43 were further tested for susceptibility to ampicillin (10 µg disc^−1^), gentamicin (10 µg disc^−1^) and chloramphenicol (30 µg disc^−1^) using disc diffusion assays on MA plates. Briefly, MA plates were inoculated with 200 µl of 5-day-old cultures of the four strains previously grown in half-strength MB, and discs were impregnated with antibiotic solutions. Strains NE40^T^, NE35, NE41 and NE43 were all resistant to gentamicin but susceptible to chloramphenicol and ampicillin (Tables 3 and S1).

## Chemotaxonomy

The fatty acid profiles (Table 4) and lipoquinones of strain NE40^T^ were determined using the services available at the University of Coimbra Bacteria Culture Collection. Fatty acid methyl esters were obtained from the fresh wet biomass and were separated, identified and quantified using the standard MIS Library Generation Software (Sherlock Microbial ID System, RTSBA 6 database, version 6.5) as previously described [40]. Lipoquinones were extracted from freeze-dried cells, purified by thin-layer chromatography, and separated by high-performance liquid chromatography [41]. The major cellular fatty acids (>10 % of the total fatty acid composition) of NE40^T^ were C_16:0_, summed feature 3 (C_16:1_ ω7c/C_16:1_ ω6c) and summed feature 8 (C_18:1_ ω7c/C_18:1_ ω6c) (Table 4). The major respiratory quinones were Q-9 and Q-8.

**Table 4.** Cellular fatty acid percent composition of strain NE40^T^, its closest type strain relatives and the type species of the *Endozoicomonas* genus Strains: 1, NE40^T^; 2, *E. gorgoniicola* PS125^T^ [9]; 3, *E. euniceicola* EF212^T^ [9]; 4, *E. montiporae* CL-33^T^ [8]; 5, *E. elysicola* MKT 110^T^ [3]. tr, trace (<1.0%); −, not detected; nd, no data available. Summed feature 2 (C_14:0_ 3-OH/iso C_16:1_); summed feature 3 (C_16:1_ ω7c/C_16:1_ ω6c); summed feature 8 (C_18:1_ ω7c/C_18:1_ ω6c).

Fatty acid	1	2	3	4	5
Hydroxy					
3-OH C_10:0_	5.2	2.4	3.0	2.9	3.1
3-OH C_12:0_	1.5	tr	1.0	–	2.8
3-OH C_12:1_	tr	–	–	nd	–
3-OH C_14:0_	nd	–	–	nd	4.1
3-OH C_16:0_	tr	–	–	nd	nd
Saturated					
C_10:0_	tr	–	–	nd	1.0
C_12:0_	tr	tr	–	nd	6.5
C_14:0_	9.7	8.5	13.8	8.5	9.3
C_15:0_	nd	–	–	nd	tr
C_16:0_	22.0	17.1	17.1	12.0	18.9
C_18:0_	tr	–	tr	–	tr
C_19:0_	nd	–	–	nd	nd
Summed features					
2	4.1	1.0	2.8	1.5	nd
3	36.1	49.4	44.0	39.6	54.5
8	18.6	19.5	16.0	32.8	5.5

Based on the phylogenetic, phylogenomic and phenotypic results provided in this study, a novel *Endozoicomonas* species is proposed here: *Endozoicomonas lisbonensis* sp. nov. (type strain NE40^T^). The four analysed strains (NE40^T^, NE35, NE41 and NE43) belong to the same here proposed species and exhibit phenotypic features found in several formally described *Endozoicomonas* species, such as the ability to grow under mesophilic, halophilic to halotolerant and neutrophilic conditions. They are motile and show catalase and oxidase activities. The major fatty acids and respiratory quinones found in this species conform with the description of other *Endozoicomonas* species. *E. lisbonensis* also presents features that distinguish it from its closest phylogenetic relatives, including *β*-galactosidase activity and the ability to reduce nitrate to nitrite.

## Description of *Endozoicomonas lisbonensis* sp. nov.

*Endozoicomonas lisbonensis* (lis.bon.en’sis. N.L. fem. adj. *lisbonensis*, pertaining to Lisbon). Cells are facultatively anaerobic, Gram-stain-negative, rod-shaped, flagellated, motile and able to reduce nitrates to nitrites. After 3 days of incubation at 30 °C on MA, colonies are translucent cream, circular, 0.5–1.5 mm in diameter, smooth and convex with entire margins. Growth occurs at 15–37 °C (optimum, 28–32 °C), at pH 6.0–8.0 (optimum, pH 7.0–8.0) and with 1–5 % (optimum; 2–3%, w/v) NaCl. Cells possess oxidase and catalase activities. Cells can hydrolyse chitin, cellulose, xylan, agarose and aesculin, but not starch. *β*-Galactosidase activity is observed; however, indole production, arginine dihydrolase activity, urease activity and gelatine hydrolysis are not detected, and cells were unable to ferment d-glucose. Cells do not utilize d-glucose, l-arabinose, d-mannose, d-mannitol, potassium gluconate, adipic acid, trisodium citrate and phenylacetic acid but can vary in their ability to utilize *N*-acetyl-glucosamine, d-maltose, capric acid and malic acid. Cells produce siderophores to chelate ferric iron. The dominant fatty acids are summed feature 3 (C_16 : 1_ ω6*c* and/or C_16 : 1_ ω7*c*), summed feature 8 (C_18 : 1_ ω6*c* and/or C_18 : 1_ ω7*c*), C_16 : 0_ and C_14 : 0_. Minor amounts (4–5%) of C_10:0_ 3-OH and summed feature 2 (C_14:0_ 3-OH/iso C_16:1_) are also found. The major respiratory quinone is Q-9, followed by Q-8. The genomic DNA G+C content of the type strain is 49.0 mol%, and its genome size is 5.45 Mb. The type strain is NE40^T^ (=DSM 118084^T^=UCCCB 212^T^), isolated from the octocoral *Litophyton* sp. at the live coral aquarium of Oceanário de Lisboa in Portugal. The GenBank/EMBL/DDBJ accession numbers for the 16S rRNA gene sequence and whole-genome shotgun project of *E. lisbonensis* NE40^T^ are PP815993 and PRJNA1075804, respectively.

## supplementary material

10.1099/ijsem.0.006696 Table S1.

## Abbreviations

ANI: average nucleotide identity
API: analytical profile index
ASW: artificial seawater
CAS: chrome azurol S
CMFASW: calcium- and magnesium-free artificial seawater
COGs: cluster of orthologous groups
dDDH: digital DNA–DNA hybridization
JGI: Joint Genome Institute
KBase: DOE Systems Biology Knowledgebase
MA: marine agar
MB: marine broth
ML: maximum likelihood
NJ: neighbour-joining
PacBio: Pacific Biosciences
R2A: Reasoner’s 2A agar
RT: room temperature
TEM: transmission electron microscopy
