# Supplementary material for: Endozoicomonas lisbonensis sp. nov., a novel marine bacterium isolated from the soft coral Litophyton sp. at Oceanário de Lisboa in Portugal
Source: Int J Syst Evol Microbiol. 2025 Mar 5;75(3):006696. doi: 10.1099/ijsem.0.006696 (PMC11883135; doi:10.1099/ijsem.0.006696)
Supplement: Table S1. [file ijsem-75-06696-s001.pdf]

Table S1 – Phenotypic features of *E. lisbonensis* strains NE40^T, NE35, NE41, and NE43. The four strains are motile and, present catalase- and oxidase activity. These strains do not produce indole and neither show arginine dihydrolase activity, gelatine hydrolysis (protease), urease activity nor do they utilize L-arabinose. Abbreviations: +, Positive; w, weakly positive; v, variable; -, negative; FAN, facultative anaerobe; S, susceptible (>3 mm inhibition zone); R, resistant (<1 mm inhibition zone).

**Characteristics**	**NE40^T**	**NE35**	**NE41**	**NE43**
Colony pigmentation	Translucid cream	Translucid cream	Translucid brown	Translucid cream
Colony morphology	Circular, smooth and convex with entire margins	Circular, smooth and convex with entire margins	Circular, smooth and convex with entire margins	Circular, smooth and convex with entire margins
Colony diameter (mm)	0.5-1.5	0.5-1.5	0.5-1.0	0.5-1.0
Gram reaction	-	-	-	-
Cell length (µm)	1.5-3.0	2.0-3.5	1.5-2.0	1.5-2.5
Cell diameter (µm)	0.5-1.0	0.5-1.0	0.5-1.0	0.5-1.0
Temperature range for growth (ºC) (optimum)	15-37 (28-32)	15-37 (28-32)	18-37 (28-32)	15-37 (28-32)
pH range for growth (optimum)	6.0-8.0 (7.0-8.0)	6.0-8.0 (7.0-8.0)	6.0-8.0 (7.0-8.0)	6.0-8.0 (7.0-8.0)
NaCl concentration for growth (%, w/v) (optimum)	1.0-5.0 (2.0-3.0)	2.0-5.0 (2.0-3.0)	1.0-5.0 (2.0-3.0)	1.0-5.0 (2.0-3.0)
Relation to O_2	FAN	FAN	FAN	FAN
Motility	+	+	+	+
Biofilm formation	w	w	w	w
Catalase	+	+	+	+
Oxidase	+	+	+	+
Reduction of nitrate	+	+	+	+
Indole production	-	-	-	-
Glucose fermentation	-	-	-	-
Arginine di-hydrolase	-	-	-	-
Urease	-	-	-	-
β-glucosidase (aesculin hydrolysis)	+	+	+	+
Gelatine hydrolysis (protease)	-	-	-	-
β-galactosidase	+	+	+	+
**Utilization of:**				
D-glucose	-	-	-	-
L-arabinose	-	-	-	-
D-mannose	-	-	-	-
D-mannitol	-	-	-	-
N-acetyl-glucosamine	v	-	-	-
D-maltose	v	-	-	-
Potassium gluconate	-	-	-	-
Capric acid	v	-	-	-
Adipic acid	-	-	-	-
Malic acid	v	-	-	-
Trisodium citrate	-	-	-	-
Phenylacetic acid	-	-	-	-
**Antibiotic resistance:**				
Ampicillin	S	S	S	S
Gentamicin	R	R	R	R
Chloramphenicol	S	S	S	S
